# The Scope of Astrocyte Elevated Gene-1/Metadherin (AEG-1/MTDH) in Cancer Clinicopathology: A Review

**DOI:** 10.3390/genes12020308

**Published:** 2021-02-22

**Authors:** Maheen Khan, Devanand Sarkar

**Affiliations:** 1 Department of Human and Molecular Genetics, Virginia Commonwealth University, Richmond, VA 23298, USA; mkhan2@mymail.vcu.edu; 2 Department of Human and Molecular Genetics, Massey Cancer Center, VCU Institute of Molecular Medicine (VIMM), Virginia Commonwealth University, Richmond, VA 23298, USA

**Keywords:** AEG-1, MTDH, biomarker, cancer

## Abstract

Since its initial cloning in 2002, a plethora of studies in a vast number of cancer indications, has strongly established AEG-1 as a bona fide oncogene. In all types of cancer cells, overexpression and knockdown studies have demonstrated that AEG-1 performs a seminal role in regulating proliferation, invasion, angiogenesis, metastasis and chemoresistance, the defining cancer hallmarks, by a variety of mechanisms, including protein-protein interactions activating diverse oncogenic pathways, RNA-binding promoting translation and regulation of inflammation, lipid metabolism and tumor microenvironment. These findings have been strongly buttressed by demonstration of increased tumorigenesis in tissue-specific AEG-1 transgenic mouse models, and profound resistance of multiple types of cancer development and progression in total and conditional AEG-1 knockout mouse models. Additionally, clinicopathologic correlations of AEG-1 expression in a diverse array of cancers establishing AEG-1 as an independent biomarker for highly aggressive, chemoresistance metastatic disease with poor prognosis have provided a solid foundation to the mechanistic and mouse model studies. In this review a comprehensive analysis of the current and up-to-date literature is provided to delineate the clinical significance of AEG-1 in cancer highlighting the commonality of the findings and the discrepancies and discussing the implications of these observations.

## 1. Introduction

Identification of a gene that can serve as a universal clinicopathological marker for cancers has important diagnostic and prognostic utility. Astrocyte elevated gene-1 (AEG-1), also known as Metadherin (MTDH) or LYsine-RIch CEACAM1 co-isolated (LYRIC), is an oncogene that serves that purpose [1]. AEG-1 was initially cloned in primary human fetal astrocytes (PHFA) as an HIV- and TNFα-inducible gene [2,3]. At the same time, the mouse homolog was cloned as MTDH as a cell membrane protein mediating breast cancer metastasis to the lungs using an in vivo phage screening approach [4], and gene trapping technique facilitated cloning of mouse/rat homologue as 3d3/lyric as an endoplasmic reticulum (ER)/nuclear envelop protein and as a tight junction protein [5,6]. A large body of current literature clearly documents that AEG-1 is overexpressed in all types of cancers analyzed to date and it is an essential molecule for the onset and progression of cancer [1].

**Figure 1:**
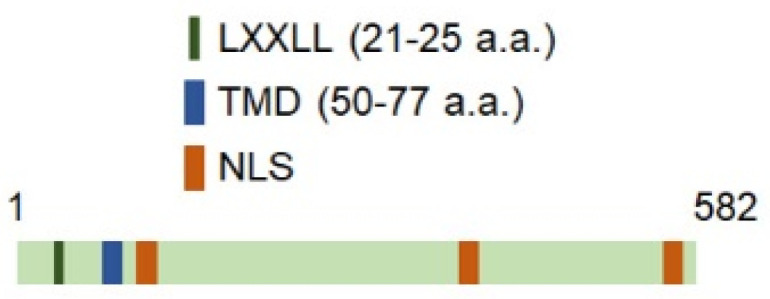
Cartoon showing AEG-1 protein not shown to scale. Human AEG-1 protein contains 582 amino acids (a.a.). TMD: Transmembrane domain facilitating anchoring in ER or cell membrane. NLS: Nuclear localization signal facilitating translocation of AEG-1 to the nucleus. LXXLL motif allows AEG-1 to interact with RXR and inhibit RXR function.

The AEG-1 locus is present on human chromosome 8q22 and contains 12 exons and 11 introns [3]. AEG-1 protein contains 582 amino acids rich in lysines, with a 50–77 amino acids single-pass transmembrane domain (TMD) and multiple nuclear localization signals (NLS) (Figure 1) [3]. It lacks any other identifiable functional or DNA-binding domains apart from an LXXLL motif present in its N-terminus (21–25 amino acids) with which AEG-1 interacts with the transcription factor Retinoid X Receptor (RXR) negatively regulating its activity [7]. The predominant intracellular localization of AEG-1 is ER-membrane [3,8,9]. In metastatic cancer cells it is also detected in the cell membrane [4]. AEG-1 is also detected in the nucleus and nucleolus, mainly in primary cells [7,10]. It has been suggested that nuclear AEG-1 is a sumoylated protein that undergoes monoubiquitination facilitating its translocation out of the nucleus and increased stability in the cytoplasm [7,10]. On the other hand it has also been shown that when stimulated by TNFα, an activator of the MAP kinase cascade and NF-κB survival pathways, AEG-1 translocates to the nucleus from the cytoplasm interacting with p65 subunit of NF-κB and CREB-binding protein (CBP), thereby augmenting NF-κB transcriptional activity [11,12]. The regulation of AEG-1 localization and its shuttling among different intracellular compartments still requires clarification.

**Figure 2:**
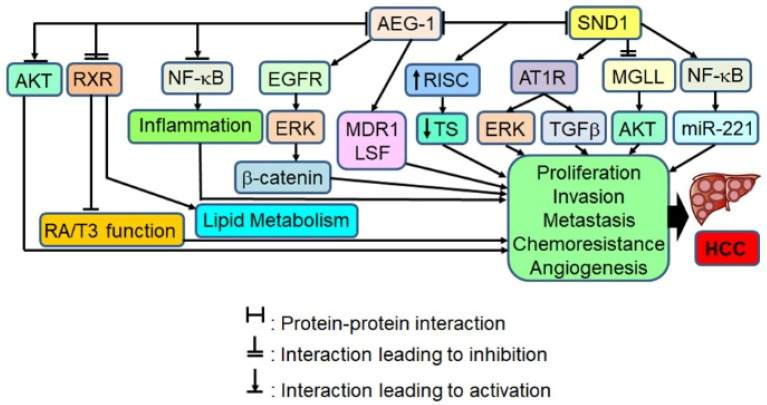
Important molecular interactions mediating AEG-1 function. AEG-1 interacts with a variety of proteins to activate oncogenic signaling pathways and modulate cellular metabolism to promote hallmarks of cancer and hence tumorigenesis. Interaction of AEG-1 with the oncogene SND1 increases RISC activity resulting in decrease in tumor suppressor genes, and activates ERK, AKT and NF-κB pathways. AEG-1 directly interacts with multiple components of NF-κB signaling pathways promoting inflammation. AEG-1 interacts with and inhibits RXR function which results in modulation of lipid metabolism and functions of vitamins and hormones. AEG-1 directly interacts with AKT to increase the duration of AKT activation. AEG-1 binds to MDR1 mRNA and increases its translation and increases the level of the transcription factor LSF contributing to chemoresistance. RXR: Retinoid X receptor, RA: Retinoic Acid, T3: thyroid hormone, EGFR: Epidermal growth factor receptor, RISC: RNA-induced silencing complex (RISC), AT1R: Angiotensin II type 1 receptor, MGLL: monoglyceride lipase, MDR1: Multiple drug resistance 1, LSF: Late SV40 factor, TS: Tumor suppressors.

AEG-1 functions as a scaffold protein and interacts with a variety of proteins and protein networks thereby activating key oncogenic pathways, such as NF-κB, PI3K/Akt, MEK/ERK, Wnt/β-catenin and TGF-β resulting in augmentation of all hallmarks of cancer, including proliferation, invasion, chemoresistance, angiogenesis and metastasis (Figure 2) [9,11,12,13,14,15,16,17]. AEG-1 displays the most significant interaction with the oncogene SND1 which is critical for mediating AEG-1 function [18,19,20]. In addition, AEG-1 also binds to specific mRNAs, especially those coding for membrane and secreted proteins, promoting their translation [8,21]. Notably, AEG-1 binds to the mRNA for Multiple drug resistance 1 (MDR1) and increases its translation which contributes to chemoresistance [22]. As a key molecule in the NF-κB signaling pathway AEG-1 plays a fundamental role in regulating inflammation, and by inhibiting the function of RXR it plays a critical role in regulating lipid metabolism and tumor microenvironment [9,11,12,17,23,24,25,26,27,28,29].

All tissues ubiquitously express AEG-1 mRNA, although skeletal muscle, heart and endocrine gland tissues have higher basal expression [3]. Compared to normal tissues and cells AEG-1 levels are markedly higher in in all spectrum of cancers analyzed to date [3]. A plethora of mechanisms dictate AEG-1 overexpression in cancers which include gene amplification, transcriptional regulation by c-Myc and NF-κB, posttranscriptional regulation by a variety of miRNAs and post-translational regulation by mono-ubiquitination and by cytoplasmic polyadenylation element-binding protein 1 (CPEB1) [10,13,23,30,31,32,33]. AEG-1 levels gradually increase with progression of cancer which correlates with adverse prognosis. Indeed, overexpression of AEG-1 promotes all hallmarks of cancer and inhibition of AEG-1 reverses these phenotypes and tissue-specific AEG-1 transgenic mouse display augmented tumorigenesis and AEG-1 knockout mouse show resistance to the development and progression of multiple cancer types, such as those of liver, breast, prostate and colon, indicating that AEG-1 plays a pivotal role in regulating tumorigenesis [13,20,22,24,25,27,28,34,35,36]. Here, we present a comprehensive description of AEG-1 expression profiles in different cancers and discuss its utility as a diagnostic/prognostic marker.

## 2. AEG-1 Is Useful Prognostic Marker for Non-Small Cell Lung Cancer (NSCLC)

NSCLC, including adenocarcinoma, squamous cell carcinoma and large cell carcinoma, represents approximately 80–85% of newly diagnosed lung cancers, with a majority of cases (70%) being in an advanced stage [37]. Furthermore, the 5-year survival of NSCLC is only 16%. Lung cancer was reported to be the worldwide leading cause of cancer-related deaths, accounting for 18.4% of deaths [38].

Multiple studies have explored the role of AEG-1 in NSCLC pathogenesis, metastasis and prognosis. A clear correlation between AEG-1 expression and NSCLC metastasis was documented by IHC analysis of 95 cases of NSCLC with non-lymphatic metastasis, 105 cases with lymphatic metastasis and 20 cases of matched distant metastasis obtained mostly from relapsed patients [39]. In all cancer tissues, AEG-1 expression was significantly higher compared to adjacent non-cancer tissues which showed undetectable or low level AEG-1 expression (*p* < 0.001). AEG-1 expression strongly correlated with lymph node metastasis (N classification; *p* = 0.015), distant metastasis (*p* = 0.004) and pathological differentiation (*p* = 0.027) and correlated inversely with overall survival time (*p* < 0.001; correlation coefficient −0.341) [39]. In both squamous cell carcinoma and adenocarcinoma, significant difference in survival time between low and high AEG-1 expression groups was observed in poorly differentiated cases (*p* < 0.001), but not in well-differentiated cases. AEG-1 was identified as an independent prognostic factor for patient outcome in univariate and multivariate analyses [39].

Primary normal lung epithelial cells demonstrated weak protein and mRNA expression of AEG-1 when compared to lung cancer cell lines in Western blot, real-time PCR and immunofluorescence assays [40]. It was observed that eight lung cancer samples showed AEG-1 overexpression in the cancerous tissues when compared to the paired normal tissues in both IHC staining and Western blot. Additional IHC analysis for AEG-1 was performed in FFPE sections of 67 NSCLC patients including 27 squamous cell carcinomas and 40 adenocarcinomas, 32 of which contained corresponding normal lung tissue [40]. Cytoplasmic high AEG-1 expression was detected in 68.7% cases especially with poor differentiation or lymph node metastases. AEG-1 expression correlated with clinical staging (*p* = 0.048), degree of differentiation (*p* = 0.023) and lymph node metastases (*p* = 0.032) as well as with MMP-2 (*p* = 0.121) and MMP-9 (*p* < 0.001) levels and inversely with overall survival (*p* < 0.001). In high and low AEG-1 expressing groups cumulative 5-year survival rate was 4.8% and 46.4%, respectively [40].

Zhang et al. sought to elucidate the clinical significance of AEG-1 in NSCLC by using IHC in tissue microarray containing 339 NSCLC and 30 normal lung tissues, and by analysis of The Cancer Genome Atlas (TCGA) database, and meta-analysis of published literature [41]. In 50.7% of NSCLC patients, AEG-1 showed high expression (*p* = 0.004) having positive correlation with clinical stage (*r* = 0.164, *p* = 0.002), lymph node metastasis (*r* = 0.232, *p* < 0.001) and tumor size (*r* = 0.240, *p* < 0.001) [41]. These findings were observed both for adenocarcinoma and squamous cell carcinoma and TCGA database analysis and meta-analysis revealed similar correlations further establishing AEG-1 as a diagnostic and prognostic marker for NSCLC [41].

IHC analysis in 225 NSCLC samples and 42 adjacent normal lung tissues showed increased AEG-1 expression in cancers (*p* < 0.001) with lymph node metastasis (*p* = 0.028) [42]. AEG-1 was identified as an independent prognostic marker for overall survival (OS) and disease-free survival (DFS) in multivariate analysis. OS (*p* = 0.014) and DFS (*p* = 0.009) were longer in low AEG-1 expressing group versus high expressing group in patients who received postoperative chemotherapy [42]. Similarly, in patients receiving postoperative radiotherapy recurrence free survival was significantly shorter in high AEG-1 expressing group (*p* = 0.016) [42]. In a separate study with small group of 38 NSCLC patients, AEG-1 expression levels correlated with lymph node metastasis (*p* = 0.001), TNM stage (*p* = 0.011) and decreased OS (*p* = 0.013) [43].

Angiogenesis is the process of formation of novel blood vessels and is well-documented to be an important player in growth and metastasis of cancers including NSCLC. In 88 paired NSCLC and adjacent normal lung tissues IHC was performed for AEG-1, vascular endothelial growth factor (VEGF) and CD105 to detect intratumoral microvessel density (iMVD) [44]. While 6.8% normal tissues showed AEG-1 expression, in NSCLC cases it was 61.3% (*p* < 0.001) which showed significant association with TNM stage (*p* = 0.021), dedifferentiation (*p* = 0.034), vascular invasion (*p* = 0.035), lymph node metastasis (*p* < 0.001) and poor overall survival (*p* = 0.024) [44]. Additionally, AEG-1 levels correlated with VEGF levels (*p* < 0.001) and iMVD (*p* < 0.001) [44].

In summary, in NSCLC AEG-1 expression level correlated strongly with advanced NSCLC especially metastatic disease and served as an independent prognostic marker for poor overall survival indicating that analysis of AEG-1 by IHC might serve as a useful prognostic marker.

## 3. Female Reproductive Cancers and AEG-1

A systematic review and meta-analysis of published literature investigated the association of AEG-1 expression with tumor metastasis and survival outcomes [45]. This analysis revealed that high AEG-1 expression significantly correlated with higher mortality and metastasis in breast, ovarian and cervical cancers [45]. In addition, polymorphisms in AEG-1 gene, such as a C/T variant in exon 11 or a G>A variant in exon 9, have been shown to confer susceptibility to breast and ovarian cancers in women from specific ethnic groups in China, which needs to be validated in more extensive studies in other patient cohorts [46,47]. Here, we provide a detailed break-down of clinicopathologic correlation of AEG-1 with each types of female reproductive cancers.

### 3.1. Breast Cancer

Among new cases, breast cancer accounts for 11.6% of all cancer cases globally (second only to lung cancer) [38,48]. Additionally, breast cancer accounts for 6.6% of cancer-related deaths [38]. Breast cancer has a particularly high burden in females, and it is the most commonly diagnosed cancer and the leading cause of cancer mortality for the female population [48]. Although chemotherapy, radiotherapy and endocrine therapy have improved overall survival, there are still obstacles in treating the invasive and metastatic nature of the disease.

DNA microarray analysis of 117 breast cancer patients identified a cluster of 4968 significant genes associated with poor prognosis among which AEG-1 was the 25th most correlated gene [49]. Using 4T1 mouse breast cancer model, AEG-1 was identified to be a cell membrane protein harboring an extracellular lung homing domain mediating breast cancer metastasis to the lungs [4]. This study also showed increased AEG-1 expression in clinical breast cancer samples compared to normal breast tissue and showed AEG-1 overexpression in brain and prostate cancers (*p* < 0.05) in SAGEmap in The Cancer Genome Anatomy Project [4].

The initial evidence for the association between breast cancer clinicopathologic features and AEG-1 expression was demonstrated by IHC analysis [50]. In 225 breast cancer samples, 93.3% were AEG-1 positive out of which 44.4% was categorized as high expression [50]. AEG-1 was minimally detected in adjacent non-cancerous tissue. AEG-1 expression significantly associated with clinical staging (*p* = 0.001), as well as T (*p* = 0.004), N (*p* = 0.026) and M (*p* = 0.001) classifications, and negatively correlated with overall survival (*p* < 0.001) [50]. In the high and low-AEG-1 expressing groups 5-year survival was 45.1% and 75.7%, respectively. However, AEG-1 expression did not correlate with age, or expression of estrogen receptor, progesterone receptor, or ERBB2 receptor [50]. Interestingly, AEG-1 was primarily located in the cytoplasm in primary tumor sections while metastatic tumor sections demonstrated AEG-1 staining primarily in the nucleus [50]. In primary normal breast epithelial cells, there was very weak detection of AEG-1 on Western blot, while seven unique breast cancer cell lines all demonstrated variable expression of AEG-1 with the highest AEG-1 expression detected at 29-fold [50]. These breast cancer patients also showed correlation between AEG-1 and proliferation marker Ki-67 (*p* = 0.003) in a subsequent study indicating that AEG-1 is associated with highly proliferative breast cancers [51]. However, knocking down AEG-1 in breast cancer cells did not affect proliferation, although there was marked inhibition in migration, invasion and metastasis, arguing against the clinicopathologic study [52].

Copy number alterations (CNA) of oncogenes is widely observed in human cancers. Using a new algorithm to analyze expression profiles of tumor specimen databases, Hu et al. detected gain of chromosome 8q22, which contains AEG-1 gene, in poor-prognosis breast cancer [52]. Kaplan–Meier curves constructed to determine the effect of 8q22 gain on survival revealed significantly (*p* < 0.05) lower metastasis- and cancer-free survival in breast cancer patients with high 8q22 gain compared with low 8q22 gain, further validating the importance of AEG-1 expression in breast cancer prognosis [52]. This finding was confirmed using Q-RT-PCR and fluorescence in situ hybridization (FISH). In 36 breast cancer specimens, 10 (27.8%) contained aberrantly high CNA at 8q22 [52]. Q-RT-PCR confirmed a strong, positive correlation between 8q22 copy numbers and AEG-1 expression. IHC analysis of 170 breast cancer patient samples revealed high AEG-1 expression in 47% cases [52]. AEG-1 expression did not correlate with any specific breast cancer subtype but significantly associated with a higher risk of metastasis (*p* = 0.0058) and shorter survival time (*p* = 0.0008) [52]. A multivariate Cox analysis showed a significant association between AEG-1 expression and metastatic hazard (*p* = 0.023) while other variables were controlled [52]. Additionally, multivariate analysis also showed that AEG-1 serves as a prognostic marker independent of other parameters, such as ER, PR, HER2 and p53 status and primary tumor size [52]. Microarray analysis of AEG-1-knockdown cells demonstrated that genes such as ALDH3A1 and MET contribute to the multidrug chemoresistance that is seen in AEG-1-positive specimens and it was hypothesized that AEG-1 may promote chemoresistance by improving survival of metastatic lesions [52].

In a subsequent study, it was documented that expression of AEG-1 was implicated in progression of precancerous breast lesions into cancerous ones [53]. Intraductal breast lesions arise from the terminal duct-lobular unit and have been organized into various subtypes, with usual ductal hyperplasia (UDH), atypical ductal hyperplasia (ADH) and ductal carcinoma in situ (DCIS) possessing various risks for progressing to invasive breast carcinoma. In 7 of 29 UDH specimens, 4 of 14 ADH specimens and 27 of 37 DCIS specimens, overexpression of AEG-1 (with localization to the cytoplasm) was identified using IHC [53]. The level of AEG-1 overexpression in all intraductal lesion types was significantly (*p* < 0.001) greater when compared to normal breast tissue [53]. When comparing levels of expression between AEG-1 and common prognostic markers of breast cancer (ER, PR and HER2), there was no significant difference in DCIS. However, there was a significant positive correlation (*p* = 0.008) between AEG-1 and Ki-67 expression in DCIS samples [53]. Furthermore, higher-graded DCIS lesions demonstrated significantly greater (*p* = 0.035) AEG-1 expression when compared to low-grade DCIS lesions suggesting that AEG-1 was overexpressed specifically in DCIS lesions that were high-graded and highly proliferative [53]. Additionally, AEG-1 overexpression was significantly associated with age (*p* = 0.042), Ki-67 status (*p* = 0.036), ER status (*p* = 0.018) and p53 status (*p* = 0.001) in invasive cancer patients, which was not observed in other previous studies [53]. AEG-1 was proposed to be a prognostic marker for precancerous ductal lesions that could be monitored for progression to invasive breast carcinoma [53].

In 125 cases of triple negative invasive breast cancer, lacking ER, PR and HER2 expression, 71 cases showed high AEG-1 expression out of which 54 cases showed high VEGF expression (*p* < 0.001) [54]. High MVD was observed in 59 cases out of which 42 displayed high AEG-1 expression (*p* = 0.002) indicating that AEG-1 is associated with increased angiogenesis [54]. Poor disease-free and overall survivals were associated with high AEG-1 and VEGF levels in Kaplan–Meier 5-year survival analysis [54]. In a recent study using tumor samples and adjacent normal breast tissue of 265 breast cancer patients, high AEG-1 and IL-10 levels showed association with poor OS (*p* = 0.0041) [55].

AEG-1 was cloned as a cell membrane protein of metastatic breast cancer cells and as such the observation that AEG-1 levels are higher in the nucleus in metastatic breast cancer cells need more in-depth analysis for validation [4,50]. IHC analysis demonstrated correlation of AEG-1 and Ki-67 levels indicating a role of AEG-1 in cell proliferation, although in vitro knock down of AEG-1 did not affect proliferation of human breast cancer cells [51,52,53]. AEG-1 levels did not correlate with ER, PR or HER2 levels indicating that AEG-1 might promote all types of breast cancers. In general, all studies confirm that AEG-1 is a useful marker for metastatic disease and poor survival. Indeed, AEG-1 is included in the FDA-approved MammaPrint early metastasis risk assessment assay for breast cancer.

### 3.2. Ovarian Cancer

Among ovarian cancers, epithelial ovarian cancer (EOC) is the most common subtype and possesses a 5-year survival of less than 30% [48]. EOC metastasizes most commonly to the peritoneal cavity which is the site of recurrence [56]. IHC of AEG-1 in 157 EOC patient samples, which include 49 lymph node metastasis and 128 peritoneal dissemination, along with 25 normal ovaries from hysterectomy patients showed little to no AEG-1 expression in normal ovaries, high AEG-1 expression in 83.7% of lymph node metastasis and in 83% with peritoneal metastasis [57]. The intensity and frequency of AEG-1 staining gradually increased from primary tumors to peritoneal metastasis to lymph node metastasis in the same patient [57]. AEG-1 expression correlated with FIGO stage (*p* = 0.0011), histopathological differentiation (*p* = 0) and residual tumor size (*p* < 0.0001) [57]. In a retrospective study of 162 specimens from patients with EOC treated with neoadjuvant chemotherapy following debulking surgery, the role of AEG-1 in promoting chemoresistance was studied [58]. Of the 162 patients, 27.2% (44) cases were non-serous ovarian carcinoma, while 72.8% (118) were serous ovarian carcinoma. The patients’ response to chemotherapy was assessed by size of the primary tumor on magnetic resonance imaging in follow-up after three cycles of neoadjuvant chemotherapy, which consisted of cisplatin/carboplatin + paclitaxel or carboplatin + docetaxel [58]. IHC analysis revealed low AEG-1 expression in 33.3% (54) of the cases, and high AEG-1 expression in 66.7% (108) of cases, the latter showing significant association with age >55 (*p* = 0.031), higher FIGO stage (*p* < 0.001), higher histologic grade (*p* < 0.001), elevated CA-125 (>35 U/mL, *p* < 0.001), residual tumor size of >1 cm (*p* < 0.001) and positive lymph node metastasis (*p* = 0.027) [58]. Fifty-seven (57) patients demonstrated chemotherapy resistance (as defined by guidelines from the Response Evaluation Criteria in Solid Tumors) out of which 84.2% (48) demonstrated AEG-1 overexpression [58]. Moreover, AEG-1 overexpression was independently associated with chemotherapy resistance on multivariate analysis (*p* = 0.026). After constructing Kaplan–Meier curves, it was found that high AEG-1 expression was negatively correlated with overall survival (*p* < 0.001) and with disease-free overall survival (*p* < 0.001) [58]. Altogether, the data pointed towards AEG-1 expression being a predictor of EOC progression and chemoresistance and suggested that AEG-1 can be useful as a therapeutic target in ovarian cancer [58].

AEG-1 has been identified to correlate with poor clinicopathologic features and outcomes of patients with stage III and IV ovarian serous carcinoma, the most common type of ovarian cancer [59]. Overexpression of AEG-1 was found to be associated with poorer overall survival and lower 5-year survival in these patients. In this study, samples from 101 patients with stage II-IV ovarian serous carcinoma were divided into either cisplatin-resistant or cisplatin-sensitive groups based on information regarding relapse or complete remission following six cycles of cisplatin chemotherapy [59]. The samples were matched against 25 normal ovarian samples obtained from patients undergoing oophorectomy or hysterectomy. In IHC normal ovarian samples demonstrated little to no AEG-1 expression, whereas AEG-1 expression was significantly upregulated in patients with chemoresistant ovarian carcinoma (*p* < 0.0001) [59]. Of note, all 32 patients identified as resistant to chemotherapy also demonstrated AEG-1 overexpression. Additionally, AEG-1 expression was stronger in cisplatin-resistant ovarian tumors versus cisplatin-sensitive tumors, indicating a potential role of AEG-1 in conferring resistance to cisplatin therapy [59]. Five-year survival curves demonstrated significantly shorter PFS (*p* < 0.001) as well as OS (*p* < 0.001) in patients with high expression of AEG-1, compared with those patients with low expression of AEG-1. In patients with high-expressing AEG-1 tumors, the median PFS was only 30.4 months, whereas PFS was 63.6 months for patients with low-expressing AEG-1 tumors [59]. Univariate analysis demonstrated that high AEG-1 expression was significantly associated with shorter OS (35.55 + 1.46 months, *p* < 0.0001) and shorter PFS (27.28 + 3.27 months, *p* < 0.0001). Notably, there was a 5-year OS of only 34.25% for patients with high AEG-1 expression [59]. Hazard ratios showed that AEG-1 overexpression, FIGO staging, residual tumor size and optimal cytoreduction were statistically significant in predicting overall survival and progression free survival. Although the exact mechanism through which AEG-1 mediated cisplatin-resistance was unidentified, thus study showed that AEG-1 may be useful in identifying those patients who are at greater risk for relapse following chemotherapy [59].

AEG-1 showed a significant correlation with poor prognosis in metastatic ovarian cancer originating from the gastrointestinal tract (e.g., stomach, colon, rectum) or breast [60]. Among 102 such patients, 77 were categorized as high AEG-1 expression, which was significantly associated with poor prognosis (*p* < 0.01) [60]. Patients with high AEG-1 expression were significantly associated with shorter median survival time in years (1.13056 for high, 2.32156 for low AEG-1 expression) and 3-year survival rates (0.1436 for high, 0.3816 for low AEG-1 expression) when compared to those of low AEG-1 expression (*p* = 0.0028) [60]. Additionally, the HRs for OS and PFS in patients with high AEG-1 expression were 2.72 (*p* = 0.0281) and 2.681 (*p* = 0.006), respectively [60]. The authors concluded that AEG-1 may not only be used as a marker for prognosis in primary ovarian cancers, but also for ovarian cancers of metastatic origin [60].

In another study, 138 patient samples of epithelial ovarian tumors were studied, with 73 epithelial ovarian cancer, 10 borderline tumor and 55 benign cystadenoma which were matched with 10 normal ovaries from patients that underwent hysterectomy [61]. With comparison to normal ovarian tissue, which showed little to no AEG-1 expression, benign cystadenomas, borderline tumors and ovarian carcinomas all stained significantly stronger for AEG-1 (*p* < 0.001) [61]. The differentiation degree by Silverberg grading, lymph node metastasis and clinical staging were significant different between low- and high-AEG-1 expression groups (*p* = 0.004, *p* = 0.009 and *p* = 0.006, respectively) [61]. AEG-1 expression was positively correlated with degree of differentiation (*p* = 0.001), lymph node metastasis (*p* = 0.008) and clinical staging (*p* = 0.002), indicating a possible association between AEG-1 overexpression and clinical progression of ovarian cancer [61]. Increased AEG-1 expression was associated with lower survival (*p* = 0.002) and AEG-1 expression was identified to be an independent prognostic factor for OS (*p* = 0.036) [61].

A clinicopathologic study unraveled a possible mechanism through which AEG-1 may promote ovarian cancer metastasis [62]. 170 patients with EOC (with 40 normal ovarian controls) were found to have concurrently elevated AEG-1 (62.9% of samples), HIF-1α (60% of samples) and VEGF (54.7% of samples) expression on IHC staining when compared to normal ovary specimens [62]. Additionally, overexpression of AEG-1 and HIF-1α were significantly higher in patients with stage III or IV ovarian carcinoma, as opposed to stage I or II (*p* < 0.001 for both) [62]. Stage I and II ovarian cancers, per the FIGO score, are characterized by confinement to the ovaries or extension below the pelvic brim in stage II. In stage III, the cancer has spread to the peritoneum or retroperitoneal lymph nodes. Stage IV ovarian cancer is characterized as distant metastasis beyond the peritoneum. Therefore, significantly increased AEG-1 protein expression in stage III and IV specimens suggests tumor metastasis beyond the pelvic brimx [62]. Subsequent molecular studies suggested a possible crosstalk between AEG-1 and the HIF-1α/NF-κB/VEGF pathways indicating the role of AEG-1 as a promoter of ovarian cancer metastasis during hypoxic conditions, which was documented in a separate study [62,63].

In summary, AEG-1 levels show highly significant correlation with peritoneal and lymph node metastases, chemoresistance and poor survival for ovarian cancer and thus, AEG-1 might be a useful diagnostic/prognostic marker for this disease.

### 3.3. Endometrial and Cervical Cancer

Endometrial cancer, a disease usually of postmenopausal women, is the most common cancer of the female genital tract [48]. It is usually diagnosed early which confers a 5-year survival rate of >80% [64]. In patients presenting with metastasis and recurrence a prognostic marker facilitates proper assessment and management of the disease. IHC analysis of 174 endometrial cancer patients and 35 healthy individuals revealed progressive increase in AEG-1 expression from normal to atypical hyperplasia to overt cancer (*p* < 0.001) [65]. Interestingly, nuclear AEG-1 staining is observed in advanced and invasive tumors. AEG-1 expression correlated with FIGO stage (*p* < 0.001), depth of myometrial invasion (*p* = 0.015), lymph node metastasis (*p* = 0.005), lymph vascular space invasion (*p* < 0.001), recurrence (*p* < 0.001) and Ki-67 expression (*p* = 0.032) [65]. Mean OS and DFS were 74 and 72 months, respectively, in low AEG-1-expressing group while they were 58 and 54 months, respectively, in high AEG-1- expressing group (*p* < 0.001) [65]. Multivariate analysis identified AEG-1 as an independent prognostic factor for poor OS and DFS [65].

Tissue microarray of 90 cervical cancer samples, including 74 squamous cervical carcinoma and 16 adenocarcinoma) demonstrated that high AEG-1 expression was significantly associated with tumor size (>4 cm, *p* = 0.010) and metastasis to lymph nodes (*p* = 0.004) [66]. There was no significant association between high AEG-1 expression and age, histologic classification, clinical stage, or pathologic grade. IHC staining of normal cervical squamous epithelium revealed absence of AEG-1, while AEG-1 staining was positive in cervical intraepithelial neoplasia (CIN) I, II and III samples, indicating AEG-1 expression may necessitate progression of CIN to cervical carcinoma [66]. Among 15 CIN III samples, 11 (73.3%) were AEG-1 positive, while only 3 of 18 (16.7%) CIN I and 6 of 17 (35.3%) CIN II samples were AEG-1 positive indicating that AEG-1 expression is associated with grading of CIN [66]. Additionally, AEG-1 staining was positive in both cervical squamous carcinoma and adenocarcinoma. High AEG-1 expression was associated with tumor size (AUC = 0.826, *p* = 0.000) and lymph node metastasis (AUC = 0.745, *p* = 0.004) and showed significantly lower OS (*p* < 0.05) compared with low AEG-1 expression [66]. High AEG-1 expression was identified as an independent prognostic factor for poor OS (hazard ratio = 4.021, *p* = 0.027) [66]. IHC staining revealed 90% of 200 cervical cancer samples stained positive for AEG-1 and AEG-1 levels showed significant correlation with clinical staging (*p* = 0.034), including T (*p* = 0.019), N (*p* = 0.038) and M classification (*p* = 0.018) and tumor differentiation (*p* = 0.043) [67]. In another study, AEG-1 overexpression was demonstrated to be greater in low-grade cervical cancers compared to high-grade cervical cancers in IHC analysis of 52 cervical cancer samples including 27 high-differentiated and 25 low-differentiated cancers, the latter showing strong upregulation of AEG-1 [68]. In vitro molecular studies revealed involvement of AEG-1 in regulating EMT, invasion and chemoresistance thereby identifying AEG-1 as a potential therapeutic target for cervical cancer [68].

The progression of cervical cancer has been attributed to microvascular proliferation, which influences invasion and metastasis and leads to poor prognosis for patients. AEG-1 was implicated as a potential inducer of microvascular proliferation in cervical cancer [69]. In 45 invasive cervical cancer samples, compared with 12 tissue samples of chronic cervicitis, AEG-1 expression was found to be significantly upregulated by IHC staining (0.186 ± 0.043, *p* < 0.01) [69]. A significant association between AEG-1 expression and vascular invasion and lymphatic metastasis (*p* < 0.01) was identified, although there was no such association between AEG-1 expression and patient age, lesion size, differentiation, pathologic subtype, or parametrial infiltration [69]. A Pearson correlation found a significant positive association between AEG-1 levels and VEGF and NF-κB levels (*p* = 0) and MVD (*p* = 0) suggesting that AEG-1 as a potential inducer of angiogenesis which could potentially contribute to invasion and metastasis by cervical cancer [69].

In general, AEG-1 levels clearly correlate with advanced metastatic disease and poor survival. However, defining the localization of AEG-1 in different stages of endometrial and cervical cancers requires more in-depth study with an AEG-1 antibody approved for clinical studies, such as Prestige Antibodies^®^ used by Human Protein Atlas.

## 4. AEG-1 and Prostate Cancer

In the US, prostate cancer is the most common cancer and the second most common cause of cancer-related deaths in males [48]. IHC analysis of AEG-1 in tissue microarray (TMA) containing 63 benign prostatic hyperplasia (BPH), 143 prostate cancer and 11 prostate cancer bone metastasis revealed that AEG-1 expression was significantly higher in prostate cancer compared to BPH (*p* = 0.037) [10]. In benign tissues, predominantly nuclear AEG-1 staining was observed and interestingly, although the tumor tissues showed low-level nuclear staining, they showed nucleolar AEG-1 staining which was not observed in benign tissues [10]. In bone metastasis, 9 out of 11 showed increased AEG-1 expression exclusively in the cytoplasm and in the cell membrane compared to normal bone. Although not statistically significant, AEG-1 expression showed a trend of higher level in patients with low Gleason score versus in patients with high Gleason score [10]. In 82.5% BPH patients, AEG-1 expression was detected in the nucleus of luminal cells and some basal cells [10]. In 26.6% prostate cancer cases (38 out of 143) nuclear AEG-1 was detected and a decrease in nuclear AEG-1 level correlated with increased Gleason score (*p* < 0.001) with reciprocal increase in cytoplasmic staining [10]. Nuclear AEG-1-positive patients had a mean survival of 70 months while in nuclear AEG-1-negative patients it was 39 months (*p* = 0.0023) [10]. Patients who did not receive any hormone treatment showed higher AEG-1 levels, irrespective of whether they responded to treatment or not, compared to patients treated with hormones. Although hormone treatment did not affect AEG-1 localization, nuclear AEG-1-positive patients showed hormone sensitivity [10]. These findings suggest that nuclear AEG-1 might protect from tumor formation, while cytoplasmic AEG-1 is more tumorigenic which is supported by studies in other cancers demonstrating a role of cytoplasmic AEG-1 in regulating miRNA function and protein translation contributing to tumorigenesis, chemoresistance and metastasis [10,21,22,23,24]. This study further indicates that in addition to expression, localization of AEG-1 might be considered a prognostic marker for cancers [10].

A separate study analyzed two TMAs composed of 62 normal prostate tissues, 10 BPH, 10 prostate atrophy or prostate inflammatory atrophy (PIA), 10 prostatic intraepithelial neoplasia (PIN), 72 prostate tumors and 10 distant metastases by IHC [34]. AEG-1 levels were undetectable or very low in all normal and BPH and gradually increased in PIN, primary tumors and metastasis with medium or high levels of expression in 10%, 47.2% and 80% cases, respectively, demonstrating a strong correlation of AEG-1 levels with clinical progression of the disease (*p* < 0.001) [34]. AEG-1 levels also correlated with Gleason score of primary tumors. Additionally, recurrence rate, determined by prostate-specific antigen (PSA) levels, was significantly higher in AEG-1-high patients compared to AEG-1-low patients [34]. Thus, AEG-1 was suggested to be a marker for prostate cancer recurrence and metastasis. Interestingly, in this study, the authors showed that knocking out AEG-1 in the transgenic adenocarcinoma of mouse prostate (TRAMP) model prolonged tumor latency and abrogated tumor progression and metastasis further establishing the importance of AEG-1 in prostate cancer [34]. However, this study did not describe AEG-1 localization to provide an opportunity to compare and contrast observation from other studies.

Although there are not a lot of clinicopathologic studies in prostate cancer, the two studies described above are in-depth and comprehensive demonstrating utility of AEG-1 as a useful diagnostic/prognostic marker for metastatic and recurrent disease with poor survival.

## 5. AEG-1 Is an Important Prognostic Marker for Gastrointestinal (GI) Cancers

The scope of AEG-1 as a clinicopathologic and prognostic biomarker in GI cancers was analyzed by a meta-analysis of published data from 2999 patients [70]. It was identified that high AEG-1 staining index was associated with tumor progression and poor survival status in all types of GI cancers thus establishing the importance of AEG-1 as a prognostic marker for GI cancers [70]. Here, we break down each type of GI cancer and highlight independent studies that analyzed AEG-1 as a clinicopathologic and prognostic marker.

### 5.1. Esophageal Cancer

Globally esophageal cancer has the seventh-highest incidence of cancer cases and is the sixth-highest in cancer-related deaths [38,71]. Esophageal cancer includes esophageal squamous cell cancer (ESCC) which is more common in Asian countries and adenocarcinoma [72]. FFPE sections from 168 ESCC patients, which include 9 stage I, 73 stage IIa, 14 stage IIb, 62 stage III and 10 stage IV cancer, were analyzed by IHC revealing 92.9% AEG-1-positive cases and AEG-1 expression was upregulated in tumor tissues compared to adjacent non-tumor tissues [73]. Spearman correlation analysis showed that AEG-1 levels statistically correlated with clinical staging (*p* = 0.001), T (*p* = 0.002), N (*p* = 0.034), M (*p* = 0.021) classifications and histological differentiation (*p* = 0.035) [73]. The cumulative 5-year survival rate in low AEG-1-expressing patients was 40.7% (95% confidence interval, 0.5095–0.3044) vs. 22.6% in high AEG-1-expressing patients (95% confidence interval, 0.3177–0.1343) [73] There was an inverse correlation between AEG-1 levels with overall survival time (*p* = 0.001) suggesting a potential of AEG-1 to serve as a prognostic marker [73]. Interestingly, male ESCC patients showed higher AEG-1 expression (*p* = 0.041) compared to female patients, the underlying molecular mechanism of which remains to be determined [73].

Residual tumor tissues were collected from 69 esophageal adenocarcinoma (EAC) patients who underwent chemoradiation treatment followed by surgery and IHC analysis revealed high AEG-1 expression in 50.7% cases (35 out of 69) [74]. Out of the 69 patients 44 patients showed relapse. AEG-1 levels did not correlate with median overall survival, type of relapse, clinical stage, tumor grade, surgical stage, percentage or residual EAC or lymphovascular invasion and the authors suggested that AEG-1 may not serve as a prognostic marker in resistant EAC after therapy [74]. This study suggests that AEG-1 might serve as a differentiating prognostic marker for ESCC and EAC, an intriguing concept that needs to be further validated in additional studies including large cohorts of both ESCC and EAC patients from different races [74].

### 5.2. Gastric Cancer

Gastric cancer is not a major cancer in the US but it is common in Asian countries and it is the fourth most common cancer globally [38,75]. IHC analysis of 105 gastric cancer patients identified overexpression of cytoplasmic AEG-1 in 66 cases. AEG-1 levels indicated advanced clinical stages as AEG-1 expression correlated with the clinical stages of the disease (*p* < 0.01) and T (*p* < 0.01), N (*p* < 0.01) and M (*p* < 0.05) classifications [76]. AEG-1 levels also correlated with proliferation marker Ki-67 (*p* < 0.01) indicating AEG-1′s role to function as a determinant of tumor growth [76]. AEG-1 overexpressing patients had a median overall 5-year survival rate of 23 months while in AEG-1-negative patients it was 38 months (*p* < 0.001). In multivariate analysis TNM stage, lymph node metastasis and AEG-1 overexpression were associated with poor overall survival [76]. AEG-1 mRNA and protein expression levels were analyzed in 119 gastric cancer patients in cancer tissues and corresponding normal adjacent mucosa identifying significant overexpression of AEG-1 in cancer tissue (*p* < 0.05) [77]. High AEG-1 mRNA and protein levels showed significant correlation with differentiation status, TNM staging, invasive depth and lymph node metastasis (*p* < 0.05) as well as overall survival [77]. These studies suggest that AEG-1 might be a prognostic marker for gastric cancer.

AEG-1 mRNA analysis in 30 paired gastric tumor and non-tumor samples from Iranian patients identified significant overexpression of AEG-1 in tumor tissue (*p* = 0.05) although the expression levels showed heterogeneity, so that 46.6% cases showed higher AEG-1 and 36.6% cases showed lower AEG-1 levels in tumor tissues compared to non-tumor tissue [78]. AEG-1 expression did not correlate with grades and types of tumor. From this study it seems that AEG-1 does not function as an important determinant for gastric cancer [78]. However, this study is limited by the analysis of AEG-1 mRNA and AEG-1 is known to be regulated by a variety of ways at the protein level, which includes monoubiquitination-mediated stabilization and regulation by CPEB1 increasing translation, that determines its overexpression [10,31]. As such, it is necessary to analyze AEG-1 protein levels in cancer patients. A potential contribution of racial variation may also underlie findings in this study.

### 5.3. Colorectal Carcinoma (CRC)

CRC accounts for approximately 1 in 10 cases of cancer, and cancer-related deaths, globally [38]. In the US in 2020, CRC is the third-leading cause of both new cancer cases as well as estimated cancer-related deaths in both sexes [48]. The clinicopathologic significance of AEG-1 in CRC has been analyzed in several studies. IHC in FFPE sections was performed in low grade adenoma (*n* = 31), high-grade adenoma (*n* = 15), colorectal carcinoma (*n* = 146) and normal colorectal mucosa (*n* = 45) as well as hepatic (*n* = 10), pulmonary (*n* = 2) and lymph node (*n* = 250) metastases [79]. Prior to surgery these patients did not receive chemo- or radiotherapy. Among the CRC patients, 42 were stage I, 38 were Stage II, 54 were stage III and 12 were stage IV based on the Union Internationale Centre le Cancer (UICC) classification [79]. Expression of AEG-1 gradually increased with progression from low-grade adenoma to high-grade adenoma to CRC showing significant correlation with UICC stage, TNM classification, Ki-67 expression and histological differentiation, while weak to no expression was detected in normal colon mucosa. [79] AEG-1 expression correlated significantly with shorter overall survival time of CRC patients (*p* < 0.001; correlation coefficient −0.38) and 5-year cumulative survival rate was 73.4% in low AEG-1 expressing group when compared to 41.5% in high AEG-1-expressing group indicating that AEG-1 might be a significant prognostic factor in CRC patients [79]. No statistical correlation was observed with age, gender, tumor location and size. Interestingly, in CRC samples and metastatic tissue overexpressed AEG-1 was detected in nucleus, with stage III/IV patients displaying higher nuclear AEG-1 staining versus stage I/II patients (46.67% vs. 24.32%, respectively; *p* = 0.037), while in normal mucosa and in adenoma it was predominantly cytoplasmic [79]. This observation is unique that in most other situations, normal cells express nuclear AEG-1 while cancer cells show cytoplasmic AEG-1 and the significance of this finding in CRC remains to be determined.

Analysis of tumor tissues, matched normal tissues and liver metastasis specimens from 520 CRC cases, which include 37 (7.12%), 204 (39.23%), 262 (50.38%) and 17 (3.27%) in Duke’s A, B, C and D clinical stages, respectively, revealed significantly higher AEG-1 expression in CRC with liver metastases [80]. Interestingly, in this study the overexpressed AEG-1 was detected in the cytoplasm and membrane, not in the nucleus [80]. AEG-1 levels positively correlated with age, Duke’s stage and distant metastasis (*p* = 0.001, 0.001 and 0.016, respectively), but not with gender and histological grade [80]. In a separate set of 56 patients, 48 (88.89%) developed liver metastasis of which 58.33% displayed AEG-1-positivity (*p* = 0.016) thus establishing AEG-1 as a determinant of CRC liver metastasis [80]. High AEG-1 expression served as an independent prognostic marker for shorter OS [80].

Another IHC-based study was performed in 120 pairs of CRC and adjacent non-tumor tissues (ANT) and 60 lymph node metastases samples [81]. In 54 CRC samples (45%) and in 13 ANT samples (10.8%) high AEG-1 expression was detected and CRC with lymph node metastasis showed higher AEG-1 expression compared to CRC without lymph node metastasis (*p* < 0.001) [81]. In 62% CRC and 0% ANT samples nuclear accumulation of AEG-1-downstream molecule β-catenin was observed with a positive statistical correlation with high AEG-1 expression (*p* < 0.001) [81].

Mean AEG-1 mRNA level was 371.56 ± 348.37 in the primary tumor and 214.98 ± 156.39 in the adjacent normal mucosa in 156 CRC patients analyzed by Q-RT-PCR [82]. In the same study, AEG-1 protein expression was analyzed by IHC in 74 distant normal colorectal mucosa, 107 adjacent normal colorectal mucosa, 158 primary CRC, 35 lymph node metastases and 9 liver metastases showing significantly higher AEG-1 protein level in primary CRC samples compared to adjacent or distant normal mucosa [82]. In lymph node metastasis a higher trend of AEG-1 expression was observed compared to primary tumors but it was not statistically significant. In CRC patients AEG-1 expressing was detected both in the nucleus and in the cytoplasm, which was significantly higher in liver metastases versus primary tumors and lymph node metastases [82]. This study, however, did not find any correlation with age, gender, location, differentiation or patient survival and AEG-1 expression level [82]. Both nuclear and cytoplasmic AEG-1 expression significantly correlated with phosphorylated NF-κB (Ser 536), p73 and Rad50 in primary tumors [82]. It was hypothesized that AEG-1 might be involved in p73-mediated DNA damage-induced apoptosis since cytoplasmic—but not nuclear—AEG-1 associated with increased apoptosis [82]. Further experimental validation is required to unravel significance of these findings. Additionally, even though it is well-established that AEG-1 is overexpressed in primary CRC tumors with further increase in the metastases, there is variability in studies in terms of correlation with other clinicopathologic parameters even when IHC was performed with the same antibody indicating that a universally accepted standard IHC protocol needs to be used. Rectal cancer patients showed higher AEG-1 expression than colon cancer patients (*p* = 0.047) and in a follow-up study of 158 rectal cancer patients treated with radiotherapy (RT), the authors documented that high AEG-1 expression in primary tumors independently correlated with higher risk of distant recurrence (*p* = 0.009) and worse DFS (*p* = 0.007), indicating that AEG-1 might be a marker to identify patients who might develop distant relapse [83].

Staphylococcal nuclease domain containing-1 (SND1) strongly interacts with AEG-1 and cooperates with AEG-1 to mediate its function [18,19,20]. In 196 CRC patients, IHC analysis revealed AEG-1 and SND1 expression in 149 (76%) and 137 (69.9%) cases, respectively, in the cancer tissue, but not in the adjacent normal tissue [84]. Among the 149 AEG-1-positive cases, 132 was positive for SND1, while among the 47 AEG-1-negative cases, 42 were SND1-negative documenting a statistically significant correlation of co-expression of these two proteins (*r* = 0.86, *p* < 0.001) [84]. AEG-1 and SND1 expression was significantly higher in aggressive nodal status (N2), late pathological stage and poor differentiation versus N0-N1 nodal status (*p* = 0.02), early pathological stage (*p* = 0.006) and moderate differentiation (*p* = 0.03), respectively [84]. No correlation was observed with age, sex or tumor status. In these patients AEG-1+ /SND1+ status was significantly associated with aggressive nodal status (*p* = 0.02), late pathological stage (*p* = 0.01), poor differentiation (*p* < 0.001) and shorter overall survival (*p* = 0.01) [84]. Overall survival in AEG-1-/SND1-cases was significantly longer versus AEG-1-/SND1+, AEG-1+ /SND1- and AEG-1+ /SND1+ cases (*p* = 0.006). AEG-1 and SND1 co-expression negatively correlated with postoperative overall survival and positively correlated with mortality (*p* = 0.009) in cox multivariate analysis, strongly indicating that AEG-1 and SND1 can serve as prognostic factors for colon cancer [84].

In a recent study comprising 86 CRC cases and 78 controls, RT-PCR analysis of serum AEG-1 mRNA levels showed significant elevation in CRC cases compared to controls (*p* < 0.001) [85]. Diagnostic accuracy of serum AEG-1 mRNA (AUC = 0.976) was significantly higher than other CRC screening markers, such as carcinoembryonic antigen (CEA), carbohydrate antigen 19.9 (CA19.9) and Fecal occult blood (FOB), and the combined accuracy of these markers (AUC = 0.741) was increased when used with serum AEG-1 mRNA (AUC = 0.820) [85]. High serum AEG-1 mRNA expression was associated with poorly differentiated histological grade, advanced tumor stage and lower survival rate [85]. AUC of AEG-1 was 0.820 for differentiating advanced versus early tumor stages. These studies identify serum AEG-1 mRNA levels as a screening tool for CRC which can increase the efficiency of other routine CRC screening markers [85].

In summary, all studies demonstrate that AEG-1 levels increase with the progression of CRC and negatively correlate with overall survival and AEG-1 protein levels as well as serum AEG-1 mRNA levels might serve as a useful diagnostic and prognostic marker for advanced CRC. However, there are discrepant observations on nuclear versus cytoplasmic localization of AEG-1 in metastatic CRC cells further stressing the necessity of using a standard IHC protocol with a clinically approved AEG-1 antibody.

### 5.4. Hepatocellular Carcinoma (HCC) and Cholangiocarcinoma

Amongst all cancers, HCC, representing >80% of primary liver cancers, is the fifth leading cause on a global scale and is the fourth-leading cause of cancer-related deaths [38]. Hepatitis B (HBV) and Hepatitis C (HCV) viral infections, aflatoxin toxicity, smoking, obesity, alcoholism and type 2 diabetes are the predominant risk factors for developing HCC. A single nucleotide polymorphism (SNP) has been identified at position -483 of AEG-1 promoter in 4 out of 53 human HCC patients but not in 108 control individuals [86]. This A>C SNP alters AP2 binding site in AEG-1 promoter and was suggested to be associated with HCC susceptibility in Iranian patients [86].

AEG-1 overexpression in HCC was established by IHC analysis in TMA containing 86 primary HCC, 23 metastatic HCC and 9 normal adjacent liver samples [13]. While very little to no AEG-1 immunostaining was detected in the normal liver samples, 93.58% of HCC samples showed variable AEG-1 levels which progressively increased with the stages I-IV and from well-differentiated to poorly differentiated (*p* < 0.0001) [13]. In a separate cohort, including 132 samples in various stages such as normal liver (*n* = 10), cirrhotic tissue (*n* = 13), low-grade dysplastic nodules (*n* = 10), high-grade dysplastic nodules (*n* = 8) and hepatocellular carcinoma (*n* = 91), AEG-1 mRNA expression was analyzed from Affymetrix microarray data. In HCV-HCC AEG-1 levels were significantly higher compared to normal and cirrhotic liver with mean up-regulation of 1.7-fold (*p* = 0.04) and 1.65-fold (*p* < 0.001), respectively [13]. Genomic amplification of AEG-1 was identified in 26% of HCC patients by DNA copy gain analysis [13]. This study did not analyze the clinicopathological correlation of AEG-1 expression in HCC patients [13]. Genomic amplification of AEG-1 in HCC patients was shown in additional studies [87].

IHC in TMA of 323 HCC patients demonstrated high AEG-1 expression in 54.2% of patients [88]. AEG-1 levels were associated with microvascular invasion (*p* < 0.001), pathologic satellites (*p* = 0.007), tumor differentiation (*p* = 0.002) and TNM stage (*p* = 0.001) but did not correlate with age, gender, liver cirrhosis, serum α-fetoprotein, tumor diameter, tumor encapsulation or BCLC stage [88]. The 1-, 3- and 5-year OS and cumulative recurrence rates were 85.4% and 25.4%, 62.2% and 50.2%, 50.7% and 59.7%, respectively [88]. Additionally, the 1-, 3- and 5-year OS in high AEG-1-expressing group were significantly lower than those in low AEG-1-expressing group (83.0% vs. 89.7%, 52.0% vs. 75.3%, 37.4% vs. 66.9%, respectively); and the 1-, 3- and 5-year cumulative recurrence rates were markedly higher in high AEG-1-expressing group than those in low AEG-1-expressing group (32.4% vs. 16.8%, 61.2% vs. 38.2%, 70.7% vs. 47.8%, respectively) [88]. AEG-1 was identified as an independent prognostic factor for both OS (HR = 1.870, *p* < 0.001) and recurrence (HR = 1.695, *p* < 0.001) by univariate and multivariate analyses [88].

IHC analysis of 85 HCC samples followed by univariate and multivariate analyses with Cox regression revealed that tumor size (HR, 2.285, 95% CI, *p* = 0.015), microvascular invasion (HR, 6.754, 95% CI, *p* = 0.008) and AEG-1 expression (HR, 4.756, 95% CI, *p* = 0.003) were independent prognostic factors for OS. [89]. For DFS tumor size (HR, 2.245, 95% CI, *p* = 0.005) and AEG-1 expression (HR, 1.916, 95% CI, *p* = 0.038) served as prognostic factors [89]. The cumulative 5-year survival and recurrence rates were 89.2% and 50.0% in low AEG-1-expressing group and 24.5% and 82.4% in high AEG-1-expressing group, respectively. Altogether, the authors concluded that AEG-1 might be a valuable prognostic factor for HCC [89]. Additional studies using IHC in TMA, TCGA database inquiry and meta-analysis of published literature reconfirmed these observations [90].

Glypican 3 (GPC-3) is a diagnostic marker for HCC and the diagnostic value of AEG-1 and GPC-3 was analyzed by IHC on HCC, adjacent nontumor tissue (ANT) and dysplastic nodules (DN) [91]. Compared to ANT and DN, in HCC both AEG-1 and GPC-3 levels were higher showing 92% and 54% positivity, respectively [91]. AEG-1 staining was found to be more diffuse while focal staining was observed for GPC-3. Alone, AEG-1 showed high sensitivity but low specificity and accuracy, while GPC-3 showed high specificity but low sensitivity and accuracy. However, combination of both augmented the sensitivity, specificity and accuracy to 94.6%, 89.5% and 90.5%, respectively, suggesting that combined AEG-1 and GPC-3 staining might facilitate early diagnosis of HCC [91].

Cholangiocarcinoma is the second most common cause of primary liver cancer after HCC. IHC analysis of 66 cases of perihilar cholangiocarcinoma (PCCA) revealed positive AEG-1 expression in 48.5% cases with higher levels in poor-differentiated group vs. well-differentiated (*p* = 0.007) and with lymph node metastasis (*p* = 0.023) [92]. However, no association was observed for age, sex, tumor diameter and tumor grade and stage. AEG-1 levels positively correlated with vimentin levels (*p* = 0.037) and negatively correlated with E-cadherin levels (*p* = 0.030) indicating association with EMT [92]. High AEG-1 levels were associated with worse OS and RFS (*p* < 0.001 and *p* = 0.01, respectively) and cox regression analysis identified that TNM stage, surgery margin and high AEG-1 expression were independent factors for OS and RFS in PCCA [92].

These clinicopathologic studies, along with studies using a variety of mouse models [24,25,27,28], have clearly documented a pivotal role of AEG-1 in driving hepatocarcinogenesis and established its utility as a diagnostic/prognostic marker for HCC.

### 5.5. Gall Bladder Cancer (GBC)

GBC is the most common cancer of the biliary tract and the fifth most common malignancy of the digestive tract and with a 5-year survival rate of 0–10% after surgery, it is one of the most lethal cancers [93]. IHC in 41 GBC, 10 adenomas and cholecystitis samples revealed gradual increase in AEG-1 staining intensity and frequency from normal mucosa to adenoma to GBC with 63.4% GBC samples showing AEG-1 overexpression (*p* = 0.0003 vs. cholecystitis) [94]. Interestingly, overexpressed AEG-1 was detected in the nucleus [94]. Increased AEG-1 expression correlated with differentiation degree (*p* = 0.0259), Nevin stage (*p* = 0.0339), liver infiltration (*p* = 0.0328) and Ki-67 expression (*p* = 0.0032), but did not correlate with age, gender, tumor location, tumor size, venous invasion, lymph node metastasis or pathological type [94]. Cox proportional hazards model identified AEG-1 as an independent prognostic factor for OS of GBC patients [94]. The mean survival time for high and low AEG-1 expressing GBC patients was 21 and 37.1 months, respectively (*p* = 0.008). The cumulative 1-, 3- and 5-year overall survival rates were 57.7%, 19.2% and 3.8% in the high AEG-1-expressing group versus 80%, 53.3% and 33.3% in low AEG-1-expressing group [94]. In this study, AEG-1 levels did not correlate with lymph node metastasis even though in most cancers AEG-1 has been documented to be a driver of metastasis. One potential explanation of this discrepancy could be that increase AEG-1 was observed in the nucleus of GBC patients, unlike other metastatic cancers where it is detected in the cytoplasm and cell membrane further stressing that the mechanism that drives AEG-1 localization requires in-depth scrutiny.

A second study aimed at more granular analysis incorporating 108 GBC patient samples, including 36 well-differentiated adenocarcinoma, 31 moderately differentiated adenocarcinomas, 30 poorly differentiated adenocarcinomas and 11 mucinous adenocarcinomas, and 96 benign samples that included 46 peritumoral tissues from the 108 GBC patients, 15 gallbladder polyps and 35 chronic cholecystitis cases [95]. Among the 46 peritumoral tissues, 10 were normal, while 10, 12 and 14 cases showed mild, moderate or severe dysplasia, respectively. Among the 35 chronic cholecystitis cases 11 were considered as normal while 12, 7 and 5 cases showed mild, moderate or severe dysplasia, respectively [95]. IHC analysis revealed significantly increased AEG-1 and EphA7 expression in GBC compared to peritumoral tissues and benign samples (*p* < 0.01), and AEG-11 and EphA7 positive benign samples showed moderate to severe dysplasia [95]. Among the 57 EphA7 positive GBC cases, 43 showed positive expression of AEG-1 while among the 51 EphA7 negative cases 32 were negative for AEG-1 (χ^2^ = 13.11, *p* < 0.001) [95]. AEG-1 and EphA7 levels were lower in well-differentiated cases with small tumor size (<2 cm), no lymph node metastasis and no invasion compared to poorly-differentiated cases with large tumor size (>2 cm), lymph node metastasis and invasion into surrounding tissues and organs (*p* < 0.05) [95]. No correlation was observed with mucinous adenocarcinoma or with sex, age or history of gallstones. Average survival time in EphA7 and AEG-1 positive cases was 8.1 months versus 13.2 months in EphA7 and AEG-1 negative cases (*p* < 0.001). Cox multivariate analysis revealed that tumor size (>2 cm), lymph node metastasis, invasion as well as AEG-1 and EphA7 expression levels were negatively correlated with postoperative survival, and positively correlated with mortality suggesting that AEG-1 and EphA7 might be prognostic factors for GBC [95]. A recent study using 71 GBC samples showed that high AEG-1 and low E-cadherin levels correlated with shorter OS and Cox multivariate analysis revealed that tumor TNM classification, histologic grade, lymphatic metastasis and AEG-1 and E-cadherin expression were independent factors for prognosis of GBC (*p* = 0.013, *p* = 0.019, *p* = 0.001, *p* = 0.011 and *p* = 0.025, respectively) [96].

It is clear from the above description that there are discrepancies in observations regarding localization of AEG-1 and its role in regulating metastasis of GBC, even though all studies concur that AEG-1 levels correlate with poor survival. More in-depth validation studies are required to clinically use AEG-1 as a diagnostic/prognostic marker for GBC.

### 5.6. Pancreatic Cancer

Pancreatic cancer is a deadly malignancy which is the fourth most common cause of cancer-related deaths in the USA and the eighth globally [38,48]. Pancreatic ductal adenocarcinoma (PDAC) is the most common type of pancreatic cancer which in most cases is diagnosed locally at an advanced stage or at metastasis with extremely poor prognosis. In 10 paired primary PDAC and adjacent non-tumor tissues, tumor/non-tumor ratio of AEG-1 mRNA was >1.5-fold in all cases as detected by Q-RT-PCR and similar finding was observed for AEG-1 protein by IHC [97]. Additionally, in 105 PDAC patients, 98.09% showed AEG-1 expression by IHC which was associated with advanced clinical stage (*p* = 0.004), T (*p* = 0.006), N (*p* = 0.003) and M (*p* = 0.007) classifications, lymph node involvement (*p* = 0.003) and distant metastasis (*p* = 0.007) [97]. No correlation was observed for age, sex, histological variant and history of alcohol consumption and tobacco smoking. High AEG-1-expressing PDAC patients had shorter OS by Kaplan–Meier analysis [97]. The cumulative 2-year survival rate was 38.09% (95% CI: 0.565–0.913) in patients with low AEG-1-expressed PDAC compared to 7.84% (95% CI: 0.403–0.697) in high AEG-1-expressed PDAC [97]. Multivariate Cox regression analysis revealed that clinical stage, T classification and AEG-1 expression were independent prognostic predictors for PDAC [97]. This single study needs to be validated by additional studies with larger cohorts.

## 6. Urinary Cancers and AEG-1

### 6.1. Renal Cell Carcinoma (RCC)

RCC is the most common tumor of adult urinary tract and comprises of ~3% of all adult cancers [98]. RCC samples from eight patients showed AEG-1 overexpression by RT-PCR and Western blot compared to matched normal kidney tissue [99]. IHC analysis in 102 RCC patients that included 86 clear cell carcinomas, 10 papillary carcinomas, 3 chromophobe cell types and three cases of cancer of the collecting duct of Bellini as well as 6 matched lymph node metastases and seven cases of neoplastic embolus in the renal vein showed high AEG-1 expression in 96 cases, especially in lymph node metastases and neoplastic emboli with weak AEG-1 expression in normal tubular epithelium and no expression in normal glomeruli [99]. Overexpressed AEG-1 was detected in the cytoplasm and correlated positively with poorly differentiated cancers [99]. AEG-1 levels correlated with clinical stage (*p* = 0.026), T classification (*p* = 0.013) and M classification (*p* = 0.032), while no correlation was observed with age, gender and N classification [99]. The cumulative 5-year survival rate was 91.3% and 52.4% in low and high AEG-1 expression groups, respectively. The mean survival time was 76.98 and 60.94 months in low and high AEG-1 expression groups, respectively, indicating a potential role of AEG-1 in regulating advanced progression of RCC [99].

IHC analysis of AEG-1 and p53 in 50 RCC patients including 24 clear cell RCC, 12 papillary RCC, 4 multilocular cystic RCC and 10 chromophobe RCC without any sarcomatoid changes identified significant association of AEG-1 levels with tumor subtypes (*p* = 0.032), tumor capsule invasion (*p* = 0.01) and lymphovascular invasion (*p* = 0.015) [100]. Similarly, p53 levels correlated with tumor diameter (*p* = 0.028), renal sinal invasion (*p* = 0.05) and Fuhrman grade (*p* = 0.026) indicating that AEG-1 and p53 might serve as prognostic markers for RCC [100]. This study did not perform combined analysis of AEG-1 and p53 and did not check whether the increased p53 is a ‘gain-of-function’ oncogenic mutant p53.

### 6.2. Bladder Cancer

In the urinary system, bladder cancer is the second most common cancer [101]. Analysis of 60 primary bladder cancer and 15 normal urothelial tissue by RT-PCR revealed high AEG-1 expression in the cancer tissues [102]. In IHC analysis, normal samples showed a staining index (SI) of 3 or less indicating negative expression while 65% of cancer samples had a SI of 4 or more [102]. For AEG-1-positive samples 77.8% belonged to the invasive (T_2_–T_4_) stage based on the Union for International Cancer Control (UICC) staging (*p* < 0.001), and 30%, 50% and 86.7% cases were in G1, G2 and G3 stages, respectively, based on WHO classification (*p* = 0.001) [102]. AEG-1 expression correlated with tumor recurrence (*p* = 0.015), Ki-67 levels (*p* < 0.001) and with multiple tumors and 82.4% multiple tumors showed positive AEG-1 expression (*p* = 0.026) [102]. In a multivariate survival analysis, patients with high SI (SI > 6) had a shorter OS compared to patients with low SI (SI < 6) (*p* < 0.001) [102]. The 5-year cumulative survival rate was 92.3% in the low SI group versus 81.3% in the high SI group suggesting that AEG-1 might be an independent prognostic marker for bladder cancer [102]. IHC analysis in non-muscle-invasive bladder cancer (NMIBC) revealed high AEG-1 expression in 45% cases showing association with tumor grade (*p* = 0.002) and progression (*p* = 0.028), and shorter PFS (*p* = 0.0011) and multivariate analysis identified AEG-1 as a prognostic factor for patients with bladder cancer [103].

Even though the findings are consistent regarding the utility of AEG-1 as a diagnostic/prognostic marker for RCC and bladder cancer more validation studies are required for clinical application of AEG-1 as a routine biomarker.

## 7. AEG-1 and Malignancy of the Nervous System

### 7.1. Neuroblastoma

Neuroblastoma, arising from peripheral neural crest, is the most common extracranial solid tumor in infants and children accounting for 7–10% of all pediatric malignancies and ~15% pediatric cancer-related deaths [104]. AEG-1 expression was significantly elevated in neuroblastoma patient-derived samples and neuroblastoma cell lines compared to normal peripheral nerve tissues and normal astrocytes [105]. IHC analysis of 32 neuroblastoma patients showed positive AEG-1 staining in all cases with 75% showing high expression, which was detected in vascular endothelial cells and glandula [106]. AEG-1 expression correlated with age (*p* = 0.012), clinical stage (*p* = 0.030) and histological stage (*p* = 0.041) and inversely correlated with OS and poor prognosis (*p* = 0.031) indicating that AEG-1 might be a potential biomarker for neuroblastoma [106].

### 7.2. Gliomas

Gliomas are rare, but frequently fatal, malignancies of the glial tissue in the central nervous system. Though rare when compared to all cancers, gliomas are the most common intracranial tumors, accounting for 81% of brain malignancies [107]. There are several glioma histological subtypes, which include (but are not limited to) anaplastic astrocytoma and glioblastoma (peak incidence at 75–84 years old), and oligodendrogliomas and oligoastrocytomas (peak incidence at 35–44 years old) [108]. Glioblastoma has the poorest survival amongst the gliomas, with an overall survival of 0.05% to 4.7% at 5 years following diagnosis [107].

IHC analysis of 296 glioma patients including 39 cases of grade 1 (13.2%), 121 cases of grade 2 (40.9%), 88 cases of grade 3 (29.7%) and 48 cases of grade 4 (16.2%) gliomas showed AEG-1 positivity in 89.5% cases, among which 48.3% were identified as low and 51.7% as high AEG-1 expression with a statistically significant difference in AEG-1 levels between normal brain and glioma samples (*p* < 0.001) [109]. Higher AEG-1 expression was detected in patients >45 years age (*p* < 0.001) and the clinicopathologic grade of the patients (*p* < 0.001) [109]. Western blot analysis of tissues from 9 normal individuals and 25 GBM, 18 astrocytomas, 18 meningiomas, 19 oligodendrogliomas and 18 other types of brain cancers revealed that compared to normal brain increased AEG-1 expression was detected in >90% glioma cases with a 3–10-fold increase, the highest being in GBM patients, which was supported by IHC analysis as well [110]. These studies also showed association with MMP-2 and MMP-9 levels with AEG-1 although other clinicopathological parameters were not analyzed [109,110]. Increased co-expression of AEG-1 and its interacting partner SND1 was observed in glioma samples which correlated with advanced grades of the disease [111].

AEG-1 interacts with Akt2 facilitating prolonged Akt2 phosphorylation and activation of downstream oncogenic signaling [14]. In primary human glioma tumor samples, AEG-1 and Akt2 were found to be upregulated when compared with normal brain tissues by IHC analysis [14]. In TCGA database among 372 patients with glioma, who were separated into high- or low-Akt2 expressing groups, high-expressing Akt2 patients demonstrated poorer OS (*p* < 0.0001) [14]. These patients were also classified into those who had either high- or low-AEG-1 expression. Patients with both low Akt2 and low AEG-1 expression were found to have the best overall clinical outcomes [14]. IHC in TMA revealed low levels of AEG-1 and Akt2 in normal brain samples and these levels showed progressive elevation in glioma samples from grade I to grade IV and a significant correlation was observed between AEG-1 and Akt2 levels in these samples (*r*^2^ = 0.9221) suggesting that AEG-1-Akt2 interaction plays a germane role in glioma pathogenesis [14].

IHC analysis of 204 tissues, including 32 normal brain tissues, 80 Low-malignant astrocytomas (LMAs) and 92 High-Malignant astrocytomas (HMAs) showed AEG-1 positivity in 74.4% tumor samples which significantly correlated with histological grades (*p* < 0.001) [112]. Additional analysis in 31 LMAs and 29 HMAs samples by RT-PCR and Western blot revealed higher AEG-1 mRNA and protein expression in HMAs than in LMAs (*p* < 0.001). AEG-1 expression did not correlate with gender or age of the patients [112]. AEG-1 interacts with MDM2 resulting in MDM2 stabilization [113] In 86 human glioma samples, IHC analysis revealed that AEG-1, MDM2 and Ki-67 expression levels progressively increased from grade II to grade IV and a positive correlation between AEG-1 and MDM2 expression was observed (*p* < 0.01) [113]. AEG-1 and MDM2 levels correlated with poor OS and pathologic stage but did not correlate with age, gender, tumor location and diameter and Cox regression analysis identified AEG-1 and MDM2 expressions as significant prognostic factor for glioma [113].

After GBM and anaplastic astrocytoma, oligodendroglioma is the 3rd most common intracranial glioma [114]. IHC analysis of 75 oligodendroglioma patients, including 52 well-differentiated and 23 anaplatic tumors, showed AEG-1 expression in 68% cases with correlated with tumor grade and Ki-67 levels (*p* = 0) but not with age and sex [115]. AEG-1 expression was hardly detected in adjacent normal brain. In high AEG-1 expressing patients, median survival time was 28 months (95% confidence interval, 25.54–30.46) and cumulative 3-year survival rate was 19.69%, while in low AEG-1 expressing patients these values were 57 months (95% confidence interval, 46.37–67.63) (*p* = 0) and 88.05%, respectively [115]. AEG-1 was identified as an independent prognostic factor for patient outcome in multivariate survival analysis [115].

Overall, in all the malignancies of the nervous system, concordant evidences support the utility of using AEG-1 as a diagnostic/prognostic marker.

## 8. Head and Neck Cancer and AEG-1

### 8.1. Salivary Gland Carcinoma (SGC)

Although SGC is a relatively rare cancer accounting for <5% of head and neck cancers it is a complex tumor with up to 24 histological subtypes [116]. IHC in FFPE sections of 141 SGC samples including nine histological subtypes, mucoepidermoid carcinoma, adenoid cystic carcinoma, acinar cell carcinoma, adenocarcinoma, squamous cell carcinoma, salivary duct carcinoma and basal cell carcinoma, detected AEG-1 positivity in 96.5% cases with weak or no staining detected in normal tissues [117]. AEG-1 expression in all types off SGC was higher than that in normal tissues with progressive increase from tumor grade I to IV (*p* < 0.001) [117]. AEG-1 levels correlated with clinical stage (*p* = 0.001), T classification (*p* = 0.008), N classification (*p* = 0.008), distant metastases (*p* = 0.006) and lymph node involvement (*p* = 0.008) [117]. However, no correlation was observed with age, gender, histological subtypes and history of drinking or smoking. High expression was associated with shorter survival time (*p* < 0.001) with a correlation coefficient of −0.383 [117]. The cumulative 5-year survival rate was 78.4% (95% CI, 0.665–0.903) in the low AEG-1 group, while it was only 45% (95% CI, 0.303–0.597) in the high AEG-1 group [117]. Although in clinical stage I-II no significant difference between low or high AEG-1 levels and OS was observed, in stages III-IV high AEG-1 levels correlated with poor survival and patients with distant metastasis and high AEG-1 levels showed shorter OS compared to patients without distant metastases [117]. AEG-1, therefore, was postulated to be a prognostic marker for late stage, aggressive SGC.

### 8.2. Head and Neck Squamous Cell Carcinoma (HNSCC)

HNSCC arises from the oral cavity, oropharynx, larynx and hypopharynx and globally it is the sixth most common malignancy [38]. Smoking, alcohol consumption and human papillomavirus (HPV) infection are some of the common causes of HNSCC [118]. IHC analysis of 20 primary HNSCC cases (oral cavity: 6, larynx: 3, oropharynx: 5, hypopharynx: 6) and corresponding normal epithelial samples revealed significantly higher AEG-1 levels in cancer tissue (*p* = 0.0154) [119]. miR-375 targets AEG-1 and its levels were significantly lower in cancer tissues (*p* = 0.008) [119]. AEG-1 is also targeted by tumor suppressor miR-30e-5p, which is downregulated in HNSCC, and low levels of miR-30e-5p correlated with poor overall survival (*p* < 0.05) [120]. IHC of 93 oral squamous cell carcinoma (OSCC) identified high AEG-1 expression in 40.86% cases mainly in the perinuclear region [121]. In low AEG-1 expressing tumors, AEG-1 positive cells were found at the peripheral cells of the tumor nests and not in the more-differentiated cells [121]. AEG-1 staining was not detected in 30 cases of normal oral mucosa. AEG-1 levels correlated with late clinical stage (*p* = 0.01) and regional lymph node metastasis (*p* < 0.001). In high and low AEG-1 expressing groups distant metastasis was observed in 10.53% and 1.83% cases, respectively and 5-year survival rate was 36.84% and 69.09%, respectively [121].

Squamous cell carcinoma of the tongue (TSCC) comprises ~41% of the oral cancers and these patients often present with lymph node metastasis [122]. IHC in 93 TSCC samples with corresponding 30 normal tongue tissue revealed AEG-1 positivity in 48.39% cases mainly in the cytoplasm with TSCC samples showing significant overexpression compared to normal tissues (*p* < 0.001) [123]. AEG-1 levels showed progressive increase from tumor grades I to IV. AEG-1 expression significantly correlated with differentiation degree (*p* < 0.001), clinical stage (*p* < 0.001), T classification (*p* = 0.007), N classification (*p* = 0.012) and lymph node involvement (*p* = 0.013). No correlation was observed with age, gender and smoking [123]. Low AEG-1 expression was significantly associated with higher overall survival (*p* = 0.004) and multivariate Cox regression analysis identified AEG-1 as an independent prognostic marker for early TSCC with well-differentiated stages, but not in moderately or poorly differentiated stages [123]. In a follow-up study analyzing 102 TSCC samples high AEG-1 levels showed correlation with high vimentin (r = 0.84) and low E-cadherin (*r* = −0.91) levels suggesting a role of AEG-1 in regulating EMT which was confirmed by subsequent molecular analysis in vitro [124]. It was demonstrated that combination of AEG-1 levels and EMT status was a reliable predictive marker of death rate. Additionally, 5-year survival rate in high AEG-1 expressing TSCC patients (16.98%, 95% CI: 32.04–50.13%) was significantly lower than low AEG-1 expressing patients (36.73%, 95% CI: 58.68–73.08%) [124].

Overall, in all types of head and neck cancers, AEG-1 levels did not correlate with precipitating risk factor, but significantly correlated with metastasis and poor overall survival indicating its potential utility as a diagnostic/prognostic marker.

## 9. Osteosarcoma and AEG-1

Osteosarcoma is the most common primary bone malignancy which is prevalent in children and adolescents [125]. IHC analysis of 62 osteosarcoma patients and 20 normal bone samples revealed 82.3% AEG-1 positivity in cancer samples out of which 32 cases showed high expression and barely detectable expression in normal bone [126]. AEG-1 levels associated with clinical stage (*r* = 0.547, *p* < 0.001), tumor classification (*r* = 0.489, *p* < 0.001), metastasis (*r* = 0.373, *p* = 0.003) and poor differentiation (*r* = 0.520, *p* < 0.001). Interestingly, AEG-1 levels were found to be higher in female patients (*r* = 0.300, *p* = 0.018) [126]. The average survival time in low AEG-1 expression group was 91.73 months (95% CI, 77.950–105.511) while that in high AEG-1 expression group was 57.188 months (95% CI, 44.608–70.308). AEG-1 was identified as an independent prognostic factor for osteosarcoma by multivariate analysis [126]. Although informative, this single study needs to be validated by other in-depth studies with larger cohorts.

## 10. Lymphoma and Leukemia and AEG-1

Diffuse large B cell lymphoma (DLBCL), an aggressive cancer of mature B lymphocytes, is the most common type of lymphoma in adults accounting for 25–50% of adult Non-Hodgkin lymphoma in the Western countries [127]. In 21 DLBCL patients and 25 patients with reactive hyperplasia of lymph nodes a significant increase (*p* < 0.0001) in AEG-1 mRNA and protein expression, analyzed by real-time PCR and Western blot, was observed in DLBCL patients compared to controls [128]. Additionally, IHC analysis of samples from 30 DLBCL patients and 15 reactive hyperplasia of the lymph nodes showed AEG-1 positivity with variable levels of expression in 76.67% DLBCL cases while little to no AEG-1 expression was detected in the controls. AEG-1 levels significantly correlated (*p* < 0.05) with the clinical staging of DLBCL patients, which was confirmed by Spearman rank correlation analysis (0.507; *p* = 0.004) but did not correlate with age, gender and patient symptoms [128]. In a follow-up study the authors showed by Kaplan–Meier analysis and Cox regression model that AEG-1 negative DLBCL patients showed better prognosis compared to AEG-1 positive patients [129]. AEG-1 expression was analyzed in lymph node biopsies from 129 T-cell Non-Hodgkin lymphoma (T-NHL) patients and 17 control individuals revealing high AEG-1 expression in T-NHL patients with barely detectable levels in normal lymph nodes (*p* < 0.01) [130]. AEG-1, LC3-II and Beclin-1 levels were found to be significantly higher in T-NHL tissues (*n* = 30) versus normal lymph nodes (*n* = 16) by Western blot, real-time PCR and IHC (*p* < 0.001), suggesting a potential role of AEG-1 in regulating autophagy [131]. No other clinicopathological parameters were analyzed in these studies.

AEG-1 mRNA overexpression was detected in chronic lymphocytic leukemia (CLL) patients which was associated with Rai stage classification, and altered levels of β2-MG and lactate dehydrogenase in serum samples [132]. At the protein level, AEG-1 overexpression was detected in 87% of CLL patients. However, no other clinicopathologic parameters were analyzed [132].

A summary of the findings of the clinicopathologic studies and utility of AEG-1 as a diagnostic/prognostic marker for different cancers are presented in Table 1.

## 11. AEG-1 Antibody as a Biomarker for Cancers

In aggressive cancers AEG-1 is detected on the cell membrane giving rise to the hypothesis that autoantibody against AEG-1 might serve as a marker for advanced disease. The lung homing domain (a.a. 381–443) of human AEG-1 was used as the antigen to detect anti-AEG-1 antibody in sera from 483 different cancer patients, including 98 breast cancer, 96 HCC, 88 colorectal cancer, 51 lung cancer and 88 gastric cancer, by ELISA [133]. At titers of ≥1:50 anti-AEG-1 antibody was detected in 49% of these patients, including 45% breast cancer, 50% HCC, 49% colorectal cancer, 45% lung cancer and 49% gastric cancer patients, with none of 230 normal individuals displaying positivity (*p* < 0.01) [133]. At this titer the antibody was detected in 59% of patients <60 years old, versus 36% patients >60 years old (*p* < 0.01) indicating that the antibody response decreases with age [133]. No difference was observed with sex or metastasis status, even though one would anticipate that metastatic tumors would express the antibody at a higher level because of cell surface expression of AEG-1. In stage I and II cancer patients, anti-AEG-1 antibody was detected in 31% cases that include 30% breast cancer, 26% HCC, 35% colorectal cancer, 30% lung cancer and 39% gastric cancer patients [133]. However, in stage III and IV cancer patients, the antibody was detected in 56% cases that include 51% breast cancer, 62% HCC, 48% colorectal cancer, 54% lung cancer and 64% gastric cancer patients (*p* < 0.01) indicating a potential use of anti-AEG-1 antibody as a marker of cancer progression [133]. Even though AEG-1 is an established diagnostic/prognostic marker for cancer, its use is limited by the availability of tumor biopsy samples and as such anti-AEG-1 antibody might serve as an important surrogate for AEG-1. However, since the original publication these findings have not been replicated by other studies thereby requiring validation in a large cohort of patients.

## 12. Conclusions

A PubMed literature search using AEG-1 or MTDH as a keyword identifies 514 papers, many of which analyzed the clinical significance of AEG-1 overexpression in cancers firmly establishing AEG-1 as a diagnostic/prognostic marker for most common cancers as well as a key determinant of metastasis. These endeavors resulted in inclusion of AEG-1 in MammaPrint early metastasis risk assessment assay (http://www.agendia.com/pages/mammaprint/21.php) which is the first and only FDA-approved individualized metastasis risk assessment assay for breast cancer that includes a unique 70-gene signature including AEG-1. However, considering that an inverse relationship exists between AEG-1 levels and OS and recurrence in all cancers studied, routine analysis of AEG-1 expression in tissue biopsies might be implemented to determine patient prognosis. AEG-1 overexpression strongly confers chemoresistance and a few clinical correlative studies have analyzed AEG-1′s role as a marker for response to chemotherapy and development of chemoresistance. However, more stringent clinical research is required to employ AEG-1 as a screening biomarker before initiating a chemotherapy-based therapeutic regiment. While availability of biopsy samples might be a limiting factor for some cancers, the observation that serum anti-AEG-1 antibody might serve as a biomarker for aggressive cancers is encouraging and needs to be validated in multiple cohorts in independent centers. It needs to be checked whether changes in anti-AEG-1 antibody titer might be used as a marker for therapy response, failure of therapy and/or disease recurrence. Recent studies have also identified serum AEG-1 mRNA as a screening biomarker for CRC patients further broadening clinical utility of AEG-1. The procedure involves a simple RT-PCR in blood samples and as such needs to be studied extensively in other cancers to use it as a routine cancer screening tool. In the absence of a small molecule inhibitor of AEG-1, additional approaches to target AEG-1 are being actively sought and evaluated which include targeted nanoparticles delivering AEG-1 siRNA, peptidomimetic disrupting AEG-1/SND1 interaction or anti-AEG-1 antibody conjugated with gold nanoparticles [23,134,135,136]. AEG-1 is a key determinant regulating inflammation and lipid metabolism thus regulating development of obesity-induced non-alcoholic steatohepatitis (NASH), a precursor to HCC [23,26]. These recent observations implicate AEG-1 in clinical conditions far beyond cancer thus opening up scope of a plethora of future exciting research.

## Figures and Tables

**Table 1:** Utility of AEG-1 is a diagnostic/prognostic marker for multiple cancers.

Type of Cancer	Summary of Observations	Utility of AEG-1 as a Diagnostic/Prognostic Marker	Reference
Non-small cell lung cancer (NSCLC)	(i) AEG-1 levels correlated with distant metastasis, angiogenesis and predicted poor survival (ii) AEG-1 is detected in the cytoplasm of metastatic cells	There is sufficient data in the literature supporting the use of AEG-1 as a useful diagnostic/prognostic marker.	[39,40,41,42,43,44]
Breast cancer	(i) AEG-1 levels correlated with distant metastasis, chemoresistance and angiogenesis, and predicted poor survival (ii) In one study AEG-1 staining was detected mainly in the nucleus of metastatic cells requiring further validation (iii) The role of AEG-1 in regulating proliferation of breast cancer cells is controversial	AEG-1 is included in FDA-approved MammaPrint early metastasis risk assessment assay for breast cancer.	[4,50,51,52,53,54,55]
Ovarian cancer	AEG-1 levels correlated with metastasis and chemoresistance and predicted poor survival	Multiple studies conclusively demonstrate that AEG-1 might be a useful diagnostic/prognostic marker for ovarian cancer, especially chemoresistant ovarian cancer.	[57,58,59,60,61,62,63]
Endometrial and cervical cancer	(i) AEG-1 levels correlated with metastasis, angiogenesis, and poor overall survival (ii) One study identified nuclear AEG-1 in advanced and invasive endometrial cancers	AEG-1 might be a useful diagnostic/prognostic marker for advanced endometrial and cervical cancer.	[65,66,67,68,69]
Prostate cancer	Cytoplasmic AEG-1 levels correlated with high Gleason score, bone metastasis, disease recurrence and poor survival	There are two in-depth and comprehensive studies supporting the use of AEG-1 as a useful diagnostic/prognostic marker.	[10,20]
Esophageal cancer	(i) In ESCC patients AEG-1 levels correlated with TNM stages and poor survival (ii) In EAC patients AEG-1 levels did not correlate with any clinico-pathological marker	It might be possible for AEG-1 to be used as a differentiating prognostic marker for ESCC and EAC. However, more in-depth validation studies are required.	[73,74]
Gastric cancer	(i) AEG-1 protein levels correlated with TNM stages and poor survival (ii) AEG-1 mRNA levels did not correlate with grades and types of cancer in Iranian patients	More studies need to be performed checking AEG-1 mRNA and protein levels in gastric cancer patients of different races to make any conclusion.	[76,78]
Colorectal cancer (CRC)	(i) AEG-1 protein levels correlated with metastatic disease and poor survival (ii) Serum AEG-1 mRNA levels correlated with advanced tumor stage and poor survival (iii) Discrepant finding about AEG-1 localization in metastatic CRC cells	AEG-1 protein in tumor sections and AEG-1 mRNA in serum might both be useful as diagnostic/prognostic marker, especially in combination with other CRC markers.	[79,80,81,82,83,84,85]
Hepatocellular carcinoma (HCC) and cholangiocarcinoma	(i) In both HCC and cholangiocarcinoma, AEG-1 levels correlated with poorly differentiated advanced disease and decreased overall and disease-free survival (ii) The sensitivity of AEG-1 as a prognostic marker for HCC increased when combined with GPC-3	In addition to clinicopathologic studies, a significant body of literature on mouse models document an essential role of AEG-1 in hepatocarcinogenesis thereby establishing strong utility of AEG-1 as a diagnostic/prognostic marker for HCC, especially in combination with other HCC markers.	[13,88,89,90,91,92]
Gallbladder carcinoma (GBC)	(i) AEG-1 levels correlated with poor survival but there is discrepant finding whether AEG-1 correlates with metastasis (ii) In some cases, AEG-1 overexpression was detected in the nucleus	There are not enough conclusive body of literature to recommend use of AEG-1 as a diagnostic/prognostic marker for GBC.	[94,95,96]
Pancreatic cancer	AEG-1 levels correlated with metastasis and poor survival	More validation studies are required	[97]
Renal cell carcinoma (RCC) and bladder cancer	AEG-1 levels correlated with distant metastasis and poor overall survival	Findings are consistent. However, more validation studies are required	[99,100,102,103]
Malignancies of the nervous system	AEG-1 levels correlated with advanced disease and poor survival	Clinicopathologic analysis supported by mechanistic studies strongly indicate the utility of AEG-1 as a diagnostic/prognostic marker for these cancers.	[14,105,106,109,110,111,112,113,115]
Head and neck cancers	(i) In all types of head and neck cancers AEG-1 levels correlated with metastasis and poor survival (ii) AEG-1 levels did not correlate with risk factors, such as smoking	Cohort sizes were large enough to establish AEG-1 as a diagnostic/prognostic marker for all types of head and neck cancers irrespective of the risk factors.	[117,119,120,121,122,123,124]
Osteosarcoma	AEG-1 levels correlated with metastasis and poor survival	More validation studies are required	[126]
Lymphomas and leukemias	AEG-1 levels were increased in these patients	More extensive studies are required	[128,129,130,131,132]
